# Extraction Behavior and Quantitative Profiling of Prenylated Flavonoids from Hops (*Humulus lupulus* L.) Under Varying Solvent Polarity, Temperature, and Cryogenic Pretreatment

**DOI:** 10.3390/molecules30244743

**Published:** 2025-12-12

**Authors:** Nora Haring, Milan Chňapek, Blažena Drábová

**Affiliations:** Faculty of Biotechnology and Food Sciences, Slovak University of Agriculture in Nitra, Tr. A. Hlinku 2, 949 76 Nitra, Slovakia; qharing@uniag.sk (N.H.); milan.chnapek@uniag.sk (M.C.)

**Keywords:** 8-prenylnaringenin (8-PN), accelerated solvent extraction (ASE), cryogenic homogenization, extraction temperature, hops (*Humulus lupulus* L.), HPLC-DAD quantification, isoxanthohumol (IXN), prenylated flavonoids, solvent polarity, xanthohumol (XN)

## Abstract

Prenylated flavonoids from hops (*Humulus lupulus* L.) represent a distinctive class of bioactive compounds with notable antioxidant and health-promoting properties. This study investigated the extraction behavior and quantitative profiles of three major prenylflavonoids—xanthohumol (XN), isoxanthohumol (IXN), and 8-prenylnaringenin (8-PN)—under varying solvent polarity (ethanol vs. methanol), extraction temperature (50–200 °C), and sample pretreatment (mechanical vs. cryogenic homogenization). Extractions were performed using accelerated solvent extraction (ASE), and compounds were quantified by HPLC-DAD. Ethanol exhibited higher extraction efficiency than methanol, while cryogenic pretreatment markedly enhanced the release of all target analytes. The maximum recovery was achieved at 150 °C for XN, 200 °C for IXN, and 100 °C for 8-PN. Multifactor statistical analysis (MANCOVA, ANOVA) confirmed significant effects of solvent, temperature, and pretreatment, as well as their interactions (*p* < 0.001). The combination of ASE and cryogenic homogenization enables efficient isolation and precise quantification of prenylated flavonoids from hops, providing a valuable analytical framework for the development of standardized hop extracts and bioactive formulations.

## 1. Introduction

The common hop (*Humulus lupulus* L.) is a rich source of secondary metabolites with a complex chemical composition, among which prenylated flavonoids represent a distinctive group of bioactive constituents. The major representatives—xanthohumol (XN), isoxanthohumol (IXN), and 8-prenylnaringenin (8-PN)—exhibit antioxidant, estrogenic, antiproliferative, and anti-inflammatory properties [1,2,3]. These compounds efficiently scavenge reactive oxygen species (ROS) and contribute to the total antioxidant capacity (TAC) of hop extracts, thereby reducing oxidative stress in biological and food systems [4,5]. Their bioactivity is closely linked to their chemical structure: substitution of the flavonoid backbone with prenyl or isoprenyl groups increases lipophilicity, stability, and membrane permeability [6]. Chemical transformations occurring during hop processing, such as the isomerization of XN into IXN or the thermal cleavage of prenyl moieties, substantially influence the distribution and abundance of these compounds in hop products and beer [7].

Prenylated flavonoids also exhibit notable antibacterial activity, further expanding their technological and biomedical relevance. Xanthohumol demonstrates strong inhibitory effects particularly against Gram-positive bacteria, including *Staphylococcus aureus*, *Streptococcus pneumoniae*, and *Enterococcus faecalis*, primarily due to its ability to disrupt bacterial membranes, impair ATP synthesis, and interfere with key redox processes [8,9]. Isoxanthohumol generally shows weaker antibacterial properties, although it retains activity against selected Gram-positive strains. In contrast, 8-prenylnaringenin exhibits moderate antibacterial potential that varies according to bacterial species and experimental conditions [10,11,12]. These documented antimicrobial effects underscore the importance of efficient extraction, stabilization, and quantitative characterization of hop prenylflavonoids.

Hops also represent a highly diverse species with dozens of cultivated varieties that differ markedly in their biochemical profiles, particularly in the content of α-bitter acids, β-bitter acids, essential oils, and prenylated flavonoids [13]. Modern breeding programs have produced aroma, dual-purpose, and high-α cultivars, each defined by characteristic concentrations of bioactive compounds. These chemical attributes are strongly influenced not only by genotype but also by geographic origin, soil composition, altitude, and climatic conditions such as temperature and photoperiod [14]. Consequently, hops grown in different regions of Europe, North America, or Oceania often exhibit substantial variability in secondary metabolites, which directly affects their extraction behavior and potential pharmacological value. This biochemical heterogeneity highlights the need for controlled and standardized experimental conditions when evaluating hop-derived compounds [15,16].

Efficient extraction of prenylated flavonoids remains challenging because these compounds occur in low concentrations and are often embedded within the resinous matrix of lupulin glands [17]. Conventional extraction techniques such as Soxhlet or maceration are time-consuming and less selective. Advanced methods, including supercritical CO_2_ extraction and accelerated solvent extraction (ASE), provide higher recovery, reduced solvent usage, and improved reproducibility [18,19,20]. Solvent polarity plays a critical role in determining prenylflavonoid solubility, with ethanol and methanol being the most commonly used extraction media; ethanol is generally preferred because of its lower toxicity and better compatibility with lipophilic structures [21,22].

The physical state of plant material also affects extraction performance. Conventional mechanical homogenization may induce degradation of thermolabile analytes, whereas cryogenic grinding minimizes oxidative and thermal alterations, thereby preserving the integrity of bioactive molecules [23]. Cryogenic lupulin separation (CLS), widely used in brewing practice, enables selective isolation of lupulin glands enriched in bitter and aromatic compounds [24]. However, its effect on the extraction efficiency and quantitative distribution of prenylated flavonoids has not yet been comprehensively evaluated.

Despite extensive research on hop polyphenols and advanced extraction techniques, the combined influence of solvent polarity, extraction temperature, and cryogenic homogenization on the quantitative recovery of prenylated flavonoids has not been systematically evaluated [25,26]. Previous ASE-based studies typically examined only one or two parameters in isolation or focused primarily on bulk phenolics, whereas the present work integrates all three key factors within a single multifactorial design. Moreover, the impact of cryogenic pretreatment on the extraction behavior and thermal stability of XN, IXN, and 8-PN has not been previously investigated. By providing a comprehensive assessment of the interactions among these variables, this study offers new insights into the mechanistic determinants of prenylflavonoid recovery and establishes a more robust analytical framework for optimizing hop extraction protocols [27,28,29].

The present study examines the extraction behavior and quantitative profiles of XN, IXN, and 8-PN from hop cones under varying solvent polarity (ethanol vs. methanol), extraction temperature (50–200 °C), and sample pretreatment (mechanical vs. cryogenic homogenization). Extractions were performed using accelerated solvent extraction coupled with HPLC-DAD quantification. The findings provide detailed insight into how solvent properties, temperature, and cryogenic pretreatment modulate the recovery and stability of prenylated flavonoids and offer a robust analytical framework for characterizing hop-derived bioactive compounds.

## 2. Results

The present study investigated the effects of three key extraction parameters—solvent type (ethanol vs. methanol), extraction temperature (50–200 °C), and sample pretreatment (mechanical vs. cryogenic homogenization)—on the recovery of the principal hop (*Humulus lupulus* L.) prenylated flavonoids xanthohumol (XN), isoxanthohumol (IXN), and 8-prenylnaringenin (8-PN). Quantitative determination was conducted using HPLC-DAD following accelerated solvent extraction (ASE). Statistical analyses (MANCOVA and ANOVA) were applied to evaluate the significance of individual factors and their interactions on extraction efficiency.

Because of the large dataset (seven hop varieties × two solvents × four extraction temperatures × two pretreatment methods), the main text presents representative results obtained for the cultivar Polaris. Polaris was selected as the representative variety because it is a high-α hop cultivar with one of the highest natural concentrations of xanthohumol, a resin-rich lupulin profile, and highly consistent chromatographic behavior across replicates, which makes it particularly suitable for illustrating extraction trends. Comprehensive data for all varieties are provided in the Supplementary Materials (Tables S1–S3, Figures S1–S6) to ensure transparency and reproducibility.

### 2.1. General Extraction Behavior

The extraction yields of XN, IXN, and 8-PN were significantly influenced by solvent polarity, extraction temperature, and pretreatment method. The three analytes exhibited distinct thermal and polarity-dependent behaviors, as reflected in their quantitative profiles.

Ethanol consistently produced higher extraction yields than methanol, which can be attributed to its lower polarity and greater affinity for amphiphilic flavonoid structures bearing prenyl substituents [21,22]. Across all examined hop varieties, the total content of prenylated flavonoids was markedly higher in ethanol extracts. This trend is consistent with the lower dielectric constant of ethanol (ε = 24.3) compared with methanol (ε = 32.6), which enhances the solubilization of lipophilic flavonoids localized within the lupulin glands [6].

These results confirm that solvent polarity is a decisive factor governing the recovery of prenylated flavonoids from hops. Ethanol provided an optimal balance between solubility, selectivity, and compound stability. Similar trends have been reported for the extraction of phenolic and flavonoid constituents using ethanol-based systems [19,20]. Given that prenylated flavonoids contribute substantially to the total antioxidant capacity (TAC) of hop extracts, higher concentrations of XN, IXN, and 8-PN in ethanol extracts may also translate into enhanced antioxidant activity [3,4].

Overall, the quantitative trends indicate that solvent properties and extraction conditions strongly modulate the chemical composition of hop extracts. The following sections provide a detailed evaluation of the individual effects and interactions of solvent type, extraction temperature, and homogenization method on the yields of XN, IXN, and 8-PN.

### 2.2. Xanthohumol (XN)

**Figure 1:**
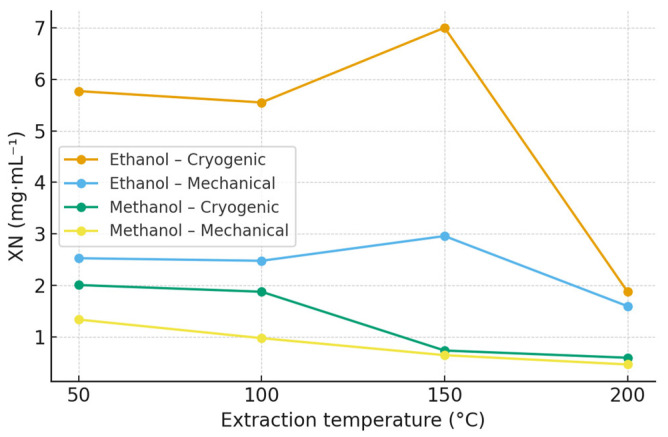
Effect of extraction temperature, solvent, and homogenization method on xanthohumol (XN) concentration in Polaris hop extracts obtained by accelerated solvent extraction (ASE). Data are expressed as mean ± SD (n = 3). Extraction was performed using ethanol and methanol at 50, 100, 150, and 200 °C. Polaris is shown as a representative variety; extraction profiles for all hop varieties are provided in the Supplementary Materials (Figure S1).

The highest concentrations of XN were obtained in ethanol extracts of the Polaris cultivar subjected to cryogenic homogenization. The maximum yield reached 7.00 mg·mL^−1^ at 150 °C, followed by 5.77 mg·mL^−1^ at 50 °C and 5.55 mg·mL^−1^ at 100 °C. In contrast, methanolic extracts showed substantially lower XN concentrations, with a maximum of 2.01 mg·mL^−1^ at 50 °C. Detailed quantitative data for XN across all hop varieties, solvents, and extraction temperatures are provided in the Supplementary Materials (Table S1; Figures S1 and S2), while extraction profiles for the Polaris cultivar are summarized in Table 1 and Figure 1.

Cryogenic pretreatment markedly enhanced XN recovery in all hop varieties, most likely due to improved disintegration of lupulin glands and increased accessibility of prenylated compounds. Extraction temperatures in the range of 100–150 °C yielded the highest XN concentrations, whereas a decline was observed above approximately 170 °C, consistent with the thermal isomerization of XN to IXN [7,30].

### 2.3. Isoxanthohumol (IXN)

**Figure 2:**
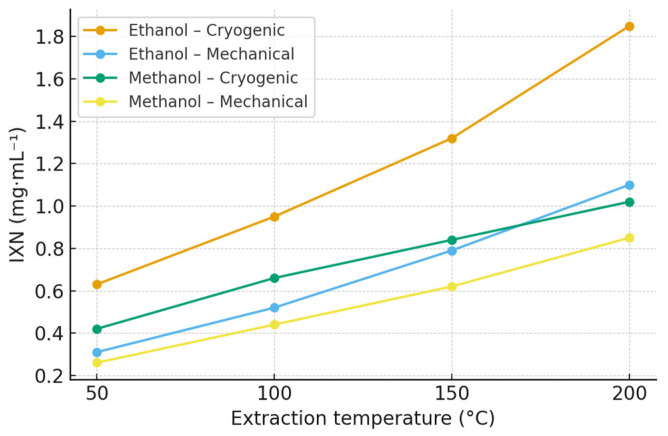
Effect of extraction temperature, solvent, and homogenization method on isoxanthohumol (IXN) concentration in Polaris hop extracts obtained by ASE. Data are expressed as mean ± SD (n = 3). Extraction was performed using ethanol and methanol at 50, 100, 150, and 200 °C. Polaris is shown as a representative variety; extraction profiles for all hop varieties are provided in the Supplementary Materials (Figure S2).

Isoxanthohumol (IXN) was primarily generated through the thermal isomerization of xanthohumol during extraction, with the highest concentrations detected at elevated temperatures. In ethanol extracts of the Polaris cultivar, the maximum IXN content reached 1.85 mg·mL^−1^ at 200 °C, whereas methanol extracts yielded a lower maximum of 1.02 mg·mL^−1^ at the same temperature. Complete quantitative data for IXN across all hop varieties and experimental conditions are provided in the Supplementary Materials (Table S2; Figures S3 and S4). Extraction profiles for the Polaris cultivar are summarized in Table 2 and Figure 2.

The observed increase in IXN content with rising temperature supports the proposed mechanism of xanthohumol isomerization under pressurized conditions [7,31]. Ethanol again yielded higher IXN concentrations than methanol, which can be attributed to its greater compatibility with prenylated structures and its lower polarity. Cryogenic homogenization further contributed to moderately higher IXN recoveries compared with mechanical processing, suggesting improved accessibility of xanthohumol precursors during the isomerization process.

### 2.4. 8-Prenylnaringenin (8-PN)

**Figure 3:**
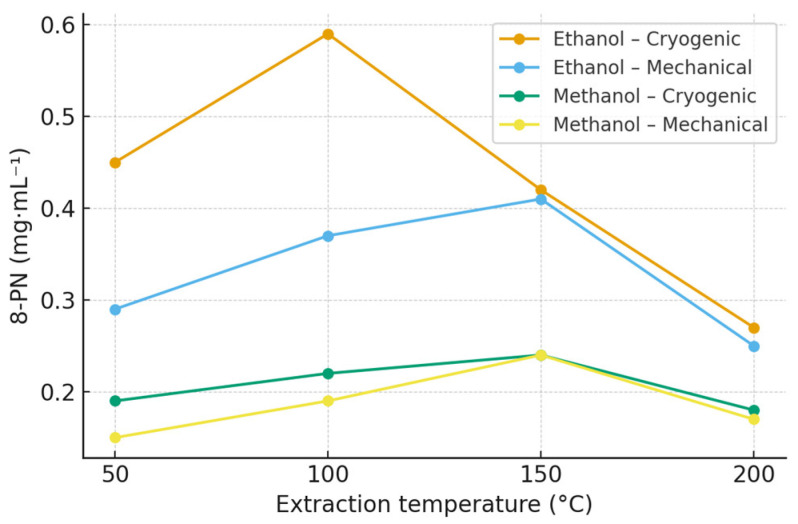
Effect of extraction temperature, solvent, and homogenization method on 8-prenylnaringenin (8-PN) concentration in Polaris hop extracts obtained by ASE. Data are expressed as mean ± SD (n = 3). Extraction was performed using ethanol and methanol at 50, 100, 150, and 200 °C. Polaris is shown as a representative variety; extraction profiles for all hop varieties are provided in the Supplementary Materials (Figure S3).

The concentrations of 8-prenylnaringenin (8-PN) were overall lower than those of xanthohumol and isoxanthohumol, which is consistent with its naturally low abundance in hop cones [32]. In ethanol extracts, the highest 8-PN yield was obtained at 100 °C (Polaris, 0.59 mg·mL^−1^), whereas higher extraction temperatures resulted in a gradual decrease in concentration, indicating possible thermal degradation or conversion into less stable derivatives [33,34]. Methanol extracts showed consistently lower yields across all temperature levels. Cryogenic homogenization enhanced the recovery of 8-PN compared with mechanical treatment, suggesting improved accessibility of bound precursors within the lupulin glands. The observed temperature-dependent decline above 150 °C supports the hypothesis of partial decomposition or structural rearrangement of prenylated naringenin derivatives under elevated thermal stress. Comprehensive quantitative data for all hop varieties and experimental conditions are provided in the Supplementary Materials (Table S3; Figures S5 and S6), and the extraction profiles for the Polaris cultivar are summarized in Table 3 and Figure 3.

### 2.5. Effect of Homogenization and Multifactor Statistical Evaluation

Cryogenic pretreatment had a consistently positive influence on the extraction of all three prenylated flavonoids. The differences between cryogenically and mechanically processed samples were statistically significant (*p* < 0.001), indicating that low-temperature grinding enhanced the accessibility of lupulin glands and improved the overall recovery of the target analytes. Multifactor ANOVA confirmed that hop variety, solvent type, extraction temperature, and homogenization method, as well as their interactions, significantly affected the quantitative yields of xanthohumol, isoxanthohumol, and 8-prenylnaringenin.

These results demonstrate that the efficiency of prenylflavonoid extraction from hops is governed by a combination of physicochemical parameters (solvent polarity, extraction temperature) and mechanical factors (sample pretreatment). Detailed statistical outputs and interaction effects are provided in the Supplementary Materials.

## 3. Discussion

**Figure 4:**
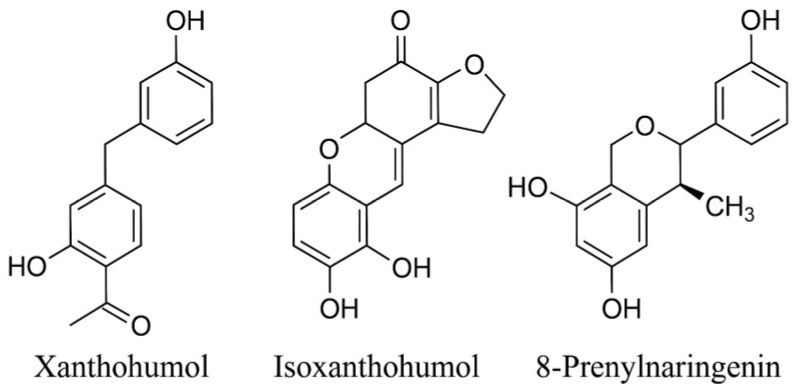
Chemical structures of the prenylated flavonoids analyzed in this study: xanthohumol (XN), isoxanthohumol (IXN), and 8-prenylnaringenin (8-PN).

The present results provide a detailed overview of how solvent polarity, extraction temperature, and cryogenic pretreatment jointly influence the recovery and quantitative distribution of the major prenylated flavonoids from *Humulus lupulus* L (Figure 4). The observed patterns are consistent with previously reported extraction behavior of hop-derived polyphenols and underscore the physicochemical factors governing prenylflavonoid solubility and stability. The following subsections interpret these trends in the context of earlier studies and discuss methodological implications as well as perspectives for future research.

### 3.1. Analytical Significance of the ASE–Cryogenic Combination

The integration of accelerated solvent extraction (ASE) with cryogenic homogenization proved effective for isolating and quantifying prenylated flavonoids from hops. This combined approach enables reproducible extraction while minimizing thermal and oxidative degradation of analytes. Enhanced solvent diffusion under pressure, together with mechanical disruption of hop tissue at low temperature, improves the accessibility of lupulin glands and promotes the release of lipophilic prenylated compounds [19,20,24]. Ethanol, an extraction solvent of intermediate polarity, exhibited the highest recovery rates, consistent with its favorable balance between polarity, solubility, and analyte stability [21]. In addition to its intermediate polarity, ethanol provides a more advantageous partitioning environment for prenylated flavonoids due to its compatibility with the lipophilic prenyl group and its enhanced ability to disrupt matrix–analyte interactions within the resin-rich lupulin glands. These properties facilitate the desorption and solubilization of XN, IXN, and 8-PN relative to methanol. Moreover, ethanol interacts effectively with residual water present in hop tissues, improving solvent penetration and promoting mass transfer under pressurized conditions. Together, these factors contribute to the superior extraction efficiency of ethanol observed in the present study [7,35,36]. Because prenylated flavonoids can undergo temperature-dependent structural transformations, particularly during high-temperature extraction, considerations of analyte stability are essential for accurate quantification.

In addition to the well-characterized isomerization of XN to IXN, high-temperature pressurized conditions may facilitate additional transformation pathways such as partial deprenylation, cyclization, or the formation of thermally induced artifacts [35,37]. Similar reactions have been reported for prenylated flavonoids and chalcones exposed to elevated thermal or oxidative stress. Minor derivatives such as 6-prenylnaringenin or isoxanthohumol epimers may also form during high-temperature extraction or prolonged thermal exposure. These compounds were not quantified in the present study because HPLC-DAD does not allow unambiguous differentiation of structurally related degradation products. Future studies employing LC-MS/MS or high-resolution mass spectrometry will be required to identify and characterize these parallel transformation pathways in greater detail [7,37,38,39].

The selected extraction temperatures also reflect the known thermochemical behavior of prenylated flavonoids under pressurized conditions. At 50–100 °C, XN, IXN, and 8-PN remain largely stable, whereas temperatures in the range of 100–150 °C have been associated with increased solubility and accelerated mass transfer of polyphenolic compounds. In contrast, temperatures approaching 200 °C have been reported to initiate the isomerization of XN to IXN and promote partial thermal degradation of prenylated structures [4,7,40]. These stability zones help contextualize the temperature-dependent extraction patterns observed in the present study.

### 3.2. Chemical and Biological Relevance of Elevated Prenylflavonoid Levels

Prenylated flavonoids such as xanthohumol and 8-prenylnaringenin are among the most biologically active hop constituents, exhibiting pronounced antioxidant, estrogenic, and anti-inflammatory effects [1,2]. Their enrichment in hop extracts is therefore not only technologically relevant but also biologically significant, as it contributes to the overall antioxidant capacity (TAC) and functional properties of hop-derived preparations [3,41,42]. Because these compounds exhibit high lipophilicity—reflected in the high logP values reported by Stevens [38] and Stevens and Page [7]—and possess strong affinity for the resinous lupulin matrix [35,43] their extraction behavior is largely governed by solvent–matrix interactions. The hydrophobic environment of lupulin glands naturally favors the partitioning of metabolites into solvents of intermediate polarity, which helps explain why ethanol yielded higher recoveries than methanol in the present study. Due to its polarity and hydrogen-bonding capacity, ethanol penetrates hop tissues more effectively and enhances the solubilization of lipophilic prenylflavonoids, ultimately resulting in improved extraction efficiency [44,45]. These findings confirm that controlled extraction parameters can modulate both the chemical composition and the functional properties of the resulting extracts.

### 3.3. Sustainable Extraction and Standardization

The ASE–cryogenic approach aligns with the principles of Green Extraction of Natural Products [46], emphasizing reduced solvent consumption, improved energy efficiency, and enhanced process reproducibility. Its capacity for precise control of temperature and solvent conditions enables the production of hop extracts with well-defined chemical profiles and consistent prenylflavonoid content, suitable for subsequent applications in food and nutraceutical formulations [47,48].

### 3.4. Broader Applicability to Other Plant Matrices

The methodological framework established here can be extended to other plant materials rich in prenylated or polyphenolic structures, including grape, citrus, soy, and various medicinal herbs [5,49,50]. The combination of ASE and cryogenic homogenization provides fine control over extraction kinetics, enabling the efficient recovery of thermolabile or low-abundance compounds while minimizing structural rearrangements and oxidative degradation [51].

### 3.5. Limitations and Future Perspectives

The present study was conducted under laboratory conditions, and scaling to pilot- or industrial-level operations should be addressed in future work [30]. Only three prenylated flavonoids were quantified, although hops contain additional derivatives (e.g., xanthohumol C, xanthohumol H, 6-prenylnaringenin) that may contribute to the overall extract composition [52,53]. Another limitation is that high-α hop cultivars generally contain a higher proportion of lupulin glands and resinous material than traditional aroma varieties such as Saaz or Premiant. These compositional differences can influence extraction kinetics and may lead to variety-specific quantitative differences in prenylflavonoid yields. Nevertheless, the multifactorial trends observed in this study—reflecting the effects of solvent type, extraction temperature, and homogenization method—remained consistent across all seven varieties examined [35,54].

In this study, several ASE parameters known to influence extraction efficiency—such as particle size, system pressure, initial moisture content, and the number of extraction cycles—were intentionally kept constant to isolate the effects of solvent polarity, temperature, and homogenization method [49]. Particle size was controlled by applying fixed mechanical and cryogenic homogenization protocols, ensuring consistent fragmentation within each treatment. Because particle size was not evaluated as an independent variable, it was maintained consistently within each homogenization approach to avoid introducing additional variability into the extraction system.

Although extraction at 200 °C enabled efficient solubilization of prenylated flavonoids, such high temperatures may also promote thermal degradation or structural rearrangements that are not fully detectable by HPLC-DAD [7,37]. Potential degradation products or early-stage transformation intermediates may remain unresolved, and future studies employing LC–MS/MS or high-resolution mass spectrometry would allow a more comprehensive characterization of these pathways. Likewise, the use of isocratic elution in the present chromatographic method provides reliable quantification of major prenylflavonoids but is not optimal for separating structurally similar minor derivatives in complex hop matrices [38,39]. Gradient LC methods would enhance chromatographic resolution and facilitate the detection of additional isomerization or degradation products [55].

Further investigations should also examine the stability of prenylflavonoids during storage and evaluate the effects of light, oxygen, and pH on compound degradation. Application of optimized extraction conditions within the framework of the circular bioeconomy could support the valorization of brewing by-products, such as brewer’s spent grain or spent hops, as alternative sources of prenylated flavonoids and polyphenols [54,56,57,58].

### 3.6. Overall Summary of the Discussion

Overall, the present work outlines a reproducible analytical framework for evaluating extraction parameters that influence prenylated flavonoids in hops. The combined ASE–cryogenic strategy, supported by ethanol as a medium-polarity solvent, provides a reliable means of isolating and quantifying bioactive constituents under controlled physicochemical conditions. These findings contribute to the development of standardized and sustainable methodologies for characterizing hop-derived bioactive compounds.

## 4. Materials and Methods

### 4.1. Plant Material

Seven hop (*Humulus lupulus* L.) varieties harvested in 2023 from different cultivation regions were used for the analysis (Table 4).

The hop varieties differed in their α- and β-bitter acid contents and in their technological purpose (aroma, bittering, or dual-purpose types). For comparison, hop material processed by cryogenic lupulin separation (CLS; variety Centennial) was also included. This technology uses liquid nitrogen to separate lupulin glands that are rich in bitter and aromatic compounds [23]. All hop materials were obtained from a certified supplier (MAROMA s.r.o., Příbram, Czech Republic), with cultivar identity confirmed based on the supplier’s documentation and origin records.

### 4.2. Sample Preparation

Homogenization was performed in two ways: (a) mechanically, by grinding 50 g of hop pellets in a porcelain mortar at room temperature; or (b) cryogenically, by freezing 50 g of pellets in 200 mL of liquid nitrogen and subsequently grinding them into a fine powder in a stainless-steel container. The pulverized samples were used immediately for extraction or stored at −80 °C.

### 4.3. Accelerated Solvent Extraction (ASE)

Extraction was performed using a Dionex ASE 350 system (Thermo Fisher Scientific, Waltham, MA, USA) at 10.5 MPa. One gram of hop sample was mixed with 2 g of diatomaceous earth (Celite) to ensure homogeneous packing of the extraction cell and to prevent channeling within the resinous lupulin matrix, thereby maintaining uniform solvent flow under pressure. Ethanol and methanol (HPLC grade, 99.9% *v*/*v*) were used as extraction solvents. Extractions were carried out at 50, 100, 150, and 200 °C in six cycles of 5 min each (total programmed extraction time 30 min) [19]. This extraction duration is in line with previously published ASE applications to hops and prenylated flavonoids, where multi-cycle programs (3–6 cycles) of 5–10 min have been reported to improve extraction completeness from resin-rich lupulin matrices [19,21,49]. The obtained extracts were evaporated under vacuum and stored at 4 °C until analysis.

### 4.4. Quantitative Analysis by HPLC-DAD

Prenylated flavonoids—xanthohumol (XN), isoxanthohumol (IXN), and 8-prenylnaringenin (8-PN)—were analyzed by high-performance liquid chromatography with diode-array detection (HPLC-DAD) using a Dionex Ultimate 3000 system (Thermo Scientific, Waltham, MA, USA). Separation was achieved on a Kromasil C18 column (250 × 4.6 mm, 5 µm) at 25 °C under isocratic conditions. The mobile phase for XN and IXN consisted of water and methanol (flow rate 1 mL·min^−1^; run time 60 min), whereas the mobile phase for 8-PN consisted of water, methanol, and isopropanol (flow rate 1 mL·min^−1^; run time 30 min). Detection wavelengths were 370 nm for XN, 290 nm for IXN, and 295 nm for 8-PN. The injection volume was 20 µL. Quantification was performed using calibration curves prepared from analytical standards (0.0625–1.0 mg·mL^−1^; R^2^ > 0.995) processed with Chromeleon 7 CDS software. All analyses were performed in triplicate.

### 4.5. Statistical Analysis

Data were evaluated using multifactor analysis of covariance (MANCOVA) and one-way ANOVA. The analyzed factors included hop variety, solvent type, homogenization method, and extraction temperature. The level of statistical significance was set at *p* < 0.05. All statistical analyses were performed using Jamovi 2.5 software (The Jamovi Project, 2023).

### 4.6. Chemicals and Analytical Standards

All chemicals and reagents were of analytical grade, and all experiments were performed in triplicate unless otherwise specified. Xanthohumol, isoxanthohumol, and 8-prenylnaringenin standards (≥98%) and solvents (ethanol, methanol, isopropanol; HPLC grade) were purchased from Sigma-Aldrich (Merck, Darmstadt, Germany). Deionized water was produced using a Milli-Q purification system (Merck Millipore, Billerica, MA, USA).

## 5. Conclusions

This study systematically evaluated the combined effects of solvent polarity, extraction temperature, and cryogenic pretreatment on the recovery of the major prenylated flavonoids from hops (*Humulus lupulus* L.). The integration of accelerated solvent extraction (ASE) with cryogenic homogenization proved to be a reliable analytical approach for isolating and quantifying xanthohumol, isoxanthohumol, and 8-prenylnaringenin. Ethanol, as a solvent of intermediate polarity, provided the highest yields while maintaining the chemical stability of the analytes.

The results demonstrate that both physicochemical (solvent and temperature) and mechanical (pretreatment) parameters significantly influence the extraction behavior and quantitative profiles of prenylated flavonoids. The proposed ASE–cryogenic workflow offers a reproducible and sustainable framework for the preparation and characterization of hop-derived bioactive compounds, supporting further development of standardized analytical and extraction protocols for natural products research.

## Figures and Tables

**Table 1:** Quantification of xanthohumol (XN) in hop extracts obtained by ASE using ethanol and methanol at different extraction temperatures (Polaris variety). Values represent mean ± SD (n = 3). Different superscript letters within the same column indicate significant differences (*p* < 0.05). ANOVA results: F (111, 224) = 929.3, *p* < 0.001; η^2^ = 0.323 (Hops), 0.266 (Extractant), 0.018 (Temperature), 0.020 (Homogenization). Polaris is shown as a representative variety; quantitative data for all hop varieties are provided in the Supplementary Materials (Table S1).

Temperature (°C)	Homogenization	Solvent	XN (mg·mL^−1^) ± SD
50	Cryogenic	Ethanol	5.77 ± 0.19 ᵃ
100	Cryogenic	Ethanol	5.55 ± 0.15 ᵃ
150	Cryogenic	Ethanol	7.00 ± 0.15 ᵃ
200	Cryogenic	Ethanol	1.88 ± 0.13 ᵇ
50	Cryogenic	Methanol	2.01 ± 0.12 ᵃ
100	Cryogenic	Methanol	1.88 ± 0.13 ᵃ
150	Cryogenic	Methanol	0.74 ± 0.06 ᵇ
200	Cryogenic	Methanol	0.60 ± 0.06 ᵇ
50	Mechanical	Ethanol	2.53 ± 0.14 ᵃ
100	Mechanical	Ethanol	2.48 ± 0.14 ᵃ
150	Mechanical	Ethanol	2.96 ± 0.13 ᵃ
200	Mechanical	Ethanol	1.60 ± 0.15 ᵇ
50	Mechanical	Methanol	1.34 ± 0.11 ᵃ
100	Mechanical	Methanol	0.98 ± 0.09 ᵇ
150	Mechanical	Methanol	0.65 ± 0.05 ᵇ
200	Mechanical	Methanol	0.47 ± 0.04 ᵇ

**Table 2:** Quantification of isoxanthohumol (IXN) in hop extracts obtained by ASE using ethanol and methanol at different extraction temperatures (Polaris variety). Values are expressed as mean ± SD (n = 3). Different superscript letters within the same column indicate significant differences (*p* < 0.05). ANOVA results: F(111, 224) = 299.5, *p* < 0.001; η^2^ = 0.291 (Hops), 0.140 (Extractant), 0.050 (Temperature), 0.034 (Homogenization). Polaris is shown as a representative variety; quantitative data for all hop varieties are provided in the Supplementary Materials (Table S2).

Temperature (°C)	Homogenization	Solvent	IXN (mg·mL^−1^) ± SD
50	Cryogenic	Ethanol	0.63 ± 0.05 ᵃ
100	Cryogenic	Ethanol	0.95 ± 0.08 ᵃ
150	Cryogenic	Ethanol	1.32 ± 0.10 ᵇ
200	Cryogenic	Ethanol	1.85 ± 0.11 ^c^
50	Cryogenic	Methanol	0.42 ± 0.04 ᵃ
100	Cryogenic	Methanol	0.66 ± 0.05 ᵇ
150	Cryogenic	Methanol	0.84 ± 0.06 ^c^
200	Cryogenic	Methanol	1.02 ± 0.07 ^c^
50	Mechanical	Ethanol	0.31 ± 0.03 ᵃ
100	Mechanical	Ethanol	0.52 ± 0.05 ᵇ
150	Mechanical	Ethanol	0.79 ± 0.06 ^c^
200	Mechanical	Ethanol	1.10 ± 0.08 ^c^
50	Mechanical	Methanol	0.26 ± 0.02 ᵃ
100	Mechanical	Methanol	0.44 ± 0.04 ᵇ
150	Mechanical	Methanol	0.62 ± 0.05 ^c^
200	Mechanical	Methanol	0.85 ± 0.06 ^c^

**Table 3:** Quantification of 8-prenylnaringenin (8-PN) in hop extracts obtained by ASE using ethanol and methanol at different extraction temperatures (Polaris variety). Values are expressed as mean ± SD (n = 3). Different superscript letters within the same column indicate significant differences (*p* < 0.05). ANOVA results: F(111, 224) = 256.7, *p* < 0.001; η^2^ = 0.174 (Extractant), 0.128 (Hops), 0.046 (Temperature), 0.082 (Homogenization). Polaris is shown as a representative variety; quantitative data for all hop varieties are provided in the Supplementary Materials (Table S3).

Temperature (°C)	Homogenization	Solvent	8-PN (mg·mL^−1^) ± SD
50	Cryogenic	Ethanol	0.45 ± 0.04 ᵃ
100	Cryogenic	Ethanol	0.59 ± 0.05 ᵇ
150	Cryogenic	Ethanol	0.42 ± 0.04 ᵇ
200	Cryogenic	Ethanol	0.27 ± 0.03 ^c^
50	Cryogenic	Methanol	0.19 ± 0.02 ᵃ
100	Cryogenic	Methanol	0.22 ± 0.02 ᵇ
150	Cryogenic	Methanol	0.24 ± 0.02 ᵇ
200	Cryogenic	Methanol	0.18 ± 0.02 ^c^
50	Mechanical	Ethanol	0.29 ± 0.03 ᵃ
100	Mechanical	Ethanol	0.37 ± 0.03 ᵇ
150	Mechanical	Ethanol	0.41 ± 0.04 ᵇ
200	Mechanical	Ethanol	0.25 ± 0.03 ^c^
50	Mechanical	Methanol	0.15 ± 0.02 ᵃ
100	Mechanical	Methanol	0.19 ± 0.02 ᵇ
150	Mechanical	Methanol	0.24 ± 0.02 ᵇ
200	Mechanical	Methanol	0.17 ± 0.02 ^c^

**Table 4:** Characteristics of the hop varieties used.

Variety	Country of Origin	α-Bitter Acids (%)	β-Bitter Acids (%)	Hopping Type
Saaz Late	Czech Republic	2.69	4.0	Second and third hopping
Premiant	Czech Republic	7.3	3.5	Universal
Centennial Cryo	USA	11.7	4.5	Second and third hopping, dry hopping
Galaxy	Australia	13.6	5.2	Third and cold hopping
Styrian Wolf	Slovenia	14.9	6.0	Second and third hopping, dry hopping
Moutere	New Zealand	15.3	7.7	Universal
Polaris	Germany	17.6	6.0	Universal

## Data Availability

The original contributions presented in this study are included in the article/Supplementary Material. Further inquiries can be directed to the corresponding author.

## Supplementary Materials

The following supporting information can be downloaded at: https://www.mdpi.com/article/10.3390/molecules30244743/s1, Figure S1: Xanthohumol extraction profile in ethanol; Figure S2: Xanthohumol extraction profile in methanol; Figure S3: Isoxanthohumol extraction profile in ethanol; Figure S4: Isoxanthohumol extraction profile in methanol; Figure S5: 8-Prenylnaringenin extraction profile in ethanol; Figure S6: 8-Prenylnaringenin extraction profile in methanol; Table S1: Full dataset of xanthohumol (XN) concentrations [mg·mL^−1^] in hop extracts obtained by accelerated solvent extraction (ASE) under different homogenization, solvent, and temperature conditions for all hop varieties; Table S2: Full dataset of isoxanthohumol (IXN) concentrations [mg·mL^−1^] in hop extracts obtained by ASE under various extraction conditions for all hop varieties; Table S3: Full dataset of 8-prenylnaringenin (8-PN) concentrations [mg·mL^−1^] in hop extracts obtained by ASE under different homogenization, solvent, and temperature conditions for all hop varieties; Table S4: Results of multifactor ANOVA for xanthohumol (XN); Table S5: Results of multifactor ANOVA for isoxanthohumol (IXN); Table S6: Results of multifactor ANOVA for 8-prenylnaringenin (8-PN).

## Abbreviations

The following abbreviations are used in this manuscript:

ASE	Accelerated Solvent Extraction
XN	Xanthohumol
IXN	Isoxanthohumol
8-PN	8-Prenylnaringenin
HPLC-DAD	High-Performance Liquid Chromatography with Diode-Array Detection
TAC	Total Antioxidant Capacity
ROS	Reactive Oxygen Species
MANCOVA	Multivariate Analysis of Covariance
ANOVA	Analysis of Variance
