# Supplementary material for: Extraction Behavior and Quantitative Profiling of Prenylated Flavonoids from Hops (Humulus lupulus L.) Under Varying Solvent Polarity, Temperature, and Cryogenic Pretreatment
Source: Molecules. 2025 Dec 12;30(24):4743. doi: 10.3390/molecules30244743 (PMC12735775; doi:10.3390/molecules30244743)
Supplement: Supplementary file 1 [file molecules-30-04743-s001.zip › molecules-4014759-supplementary.pdf]

Supplementary Materials

This supplementary file provides complete extraction datasets and statistical analyses supporting Figures 1–3 and Tables 2–4 presented in the main manuscript. The data represent mean values ± SD obtained from triplicate extractions of seven hop varieties analyzed by HPLC-DAD.

Figures S1–S6 present complete extraction profiles for xanthohumol (XN), isoxanthohumol (IXN), and 8-prenylnaringenin (8-PN) obtained with ethanol and methanol as extraction media. These figures complement the representative data shown for the Polaris variety in Figures 1–3 of the main manuscript.

Table S1. Full dataset of xanthohumol (XN) concentrations [mg·mL^{-1}] in hop extracts obtained by accelerated solvent extraction (ASE) under different homogenization, solvent, and temperature conditions for all hop varieties.

Variety	Homogenization	Solvent	50 °C	100 °C	150 °C	200 °C
Saaz Late	Mechanical	Ethanol	0.000 ± 0.000	0.023 ± 0.008	0.000 ± 0.000	0.042 ± 0.005
Saaz Late	Cryogenic	Ethanol	0.084 ± 0.000	0.051 ± 0.008	0.018 ± 0.004	0.000 ± 0.000
Premiant	Mechanical	Ethanol	0.042 ± 0.000	0.039 ± 0.008	0.010 ± 0.001	0.021 ± 0.002
Premiant	Cryogenic	Ethanol	0.002 ± 0.000	0.016 ± 0.003	0.022 ± 0.003	0.018 ± 0.002
Centennial (CSL)	Mechanical	Ethanol	0.044 ± 0.004	0.057 ± 0.012	0.033 ± 0.004	0.006 ± 0.002
Centennial (CSL)	Cryogenic	Ethanol	0.451 ± 0.013	1.180 ± 0.066	1.010 ± 0.047	0.920 ± 0.055
Galaxy	Mechanical	Ethanol	2.241 ± 0.100	1.582 ± 0.129	2.881 ± 0.115	2.285 ± 0.117
Galaxy	Cryogenic	Ethanol	2.444 ± 0.139	1.422 ± 0.097	3.354 ± 0.128	1.920 ± 0.092
Styrian Wolf	Mechanical	Ethanol	1.359 ± 0.182	1.872 ± 0.109	2.373 ± 0.111	0.026 ± 0.003
Styrian Wolf	Cryogenic	Ethanol	1.025 ± 0.131	1.392 ± 0.104	2.078 ± 0.102	0.021 ± 0.003
Moutere	Mechanical	Ethanol	2.322 ± 0.116	2.188 ± 0.115	2.782 ± 0.116	2.133 ± 0.117
Moutere	Cryogenic	Ethanol	2.857 ± 0.140	2.706 ± 0.136	3.046 ± 0.138	1.935 ± 0.094
Polaris	Mechanical	Ethanol	2.532 ± 0.111	2.475 ± 0.111	2.963 ± 0.109	1.600 ± 0.122
Polaris	Cryogenic	Ethanol	5.765 ± 0.158	5.547 ± 0.123	6.998 ± 0.125	1.877 ± 0.109

Saaz Late	Mechanical	Methanol	0.000 ± 0.000	0.013 ± 0.004	0.000 ± 0.000	0.015 ± 0.002
Saaz Late	Cryogenic	Methanol	0.049 ± 0.005	0.000 ± 0.000	0.035 ± 0.004	0.017 ± 0.002
Premiant	Mechanical	Methanol	0.012 ± 0.002	0.035 ± 0.004	0.019 ± 0.003	0.016 ± 0.002
Premiant	Cryogenic	Methanol	0.014 ± 0.002	0.000 ± 0.000	0.027 ± 0.003	0.012 ± 0.002
Centennial (CSL)	Mechanical	Methanol	0.012 ± 0.002	0.022 ± 0.003	0.014 ± 0.002	0.008 ± 0.001
Centennial (CSL)	Cryogenic	Methanol	0.068 ± 0.006	0.066 ± 0.005	0.047 ± 0.004	0.033 ± 0.003
Galaxy	Mechanical	Methanol	0.098 ± 0.008	0.084 ± 0.007	0.077 ± 0.007	0.053 ± 0.005
Galaxy	Cryogenic	Methanol	0.121 ± 0.010	0.091 ± 0.008	0.065 ± 0.006	0.044 ± 0.004
Styrian Wolf	Mechanical	Methanol	0.040 ± 0.004	0.033 ± 0.003	0.025 ± 0.003	0.018 ± 0.002
Styrian Wolf	Cryogenic	Methanol	0.029 ± 0.003	0.033 ± 0.003	0.026 ± 0.003	0.017 ± 0.002
Moutere	Mechanical	Methanol	0.023 ± 0.003	0.042 ± 0.004	0.040 ± 0.003	0.023 ± 0.003
Moutere	Cryogenic	Methanol	0.022 ± 0.002	0.000 ± 0.000	0.037 ± 0.003	0.023 ± 0.003
Polaris	Mechanical	Methanol	0.784 ± 0.051	0.935 ± 0.051	0.553 ± 0.053	0.239 ± 0.027
Polaris	Cryogenic	Methanol	2.007 ± 0.100	1.884 ± 0.107	0.739 ± 0.047	0.604 ± 0.051

Values are mean ± SD (n = 3). Different superscript letters within each column in the main text indicate significant differences ($p < 0.05$). ANOVA summary (XN): $\eta^2 = 0.323$ (Hops), 0.266 (Extractant), 0.018 (Temperature), 0.020 (Homogenization); $p < 0.001$.

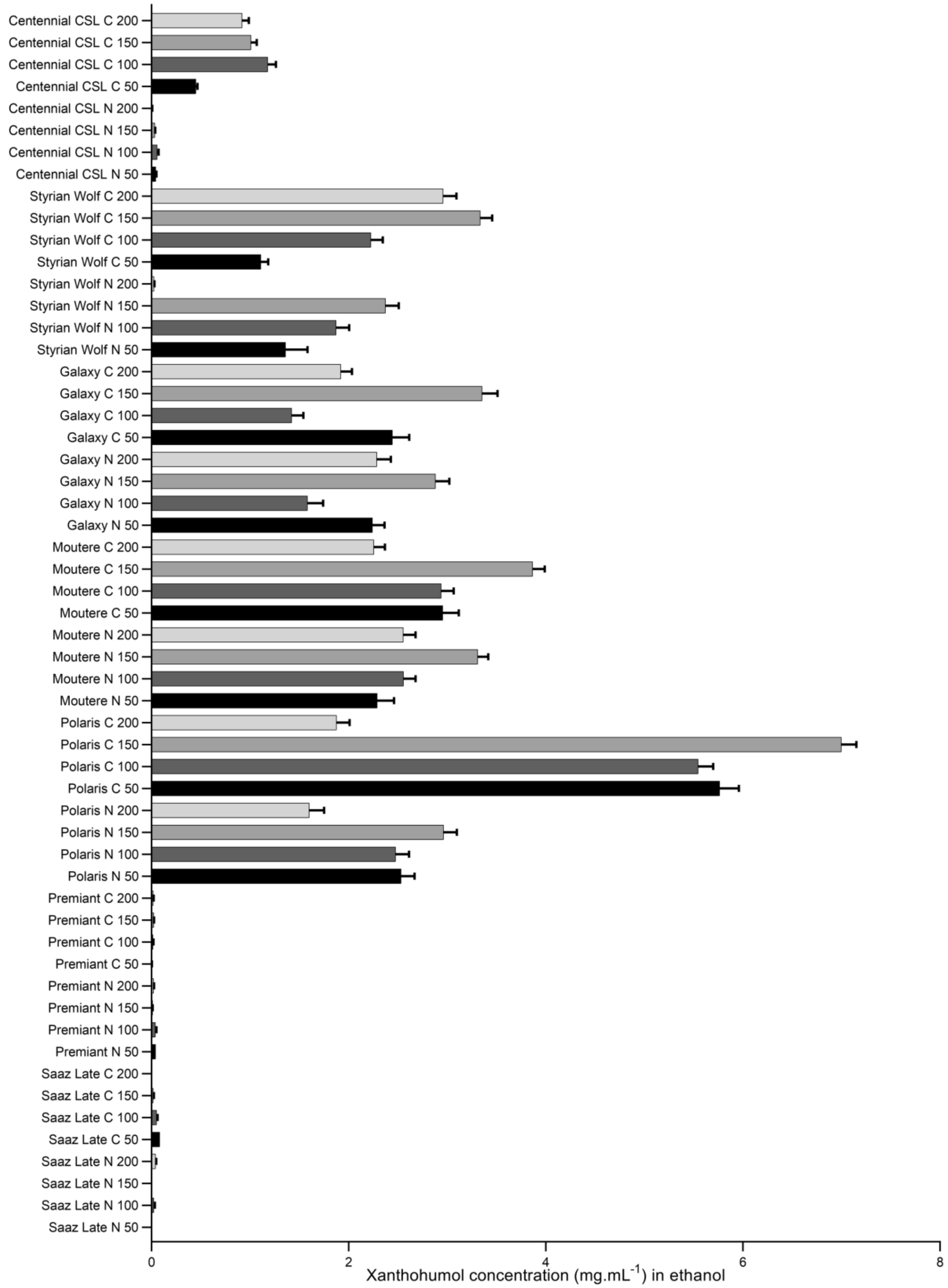

Figure S1. *Xanthohumol extraction profile in ethanol.*

Extraction profile of xanthohumol (XN) in hop extracts obtained by accelerated solvent extraction (ASE) using ethanol as the extraction medium across seven hop varieties (*Saaz Late, Premiant, Centennial Cryo, Galaxy, Styrian Wolf, Moutere, Polaris*). Data are expressed as mean ± SD (n = 3). Color intensity corresponds to extraction temperature: the darkest color represents 50 °C, while the lightest color represents 200 °C.

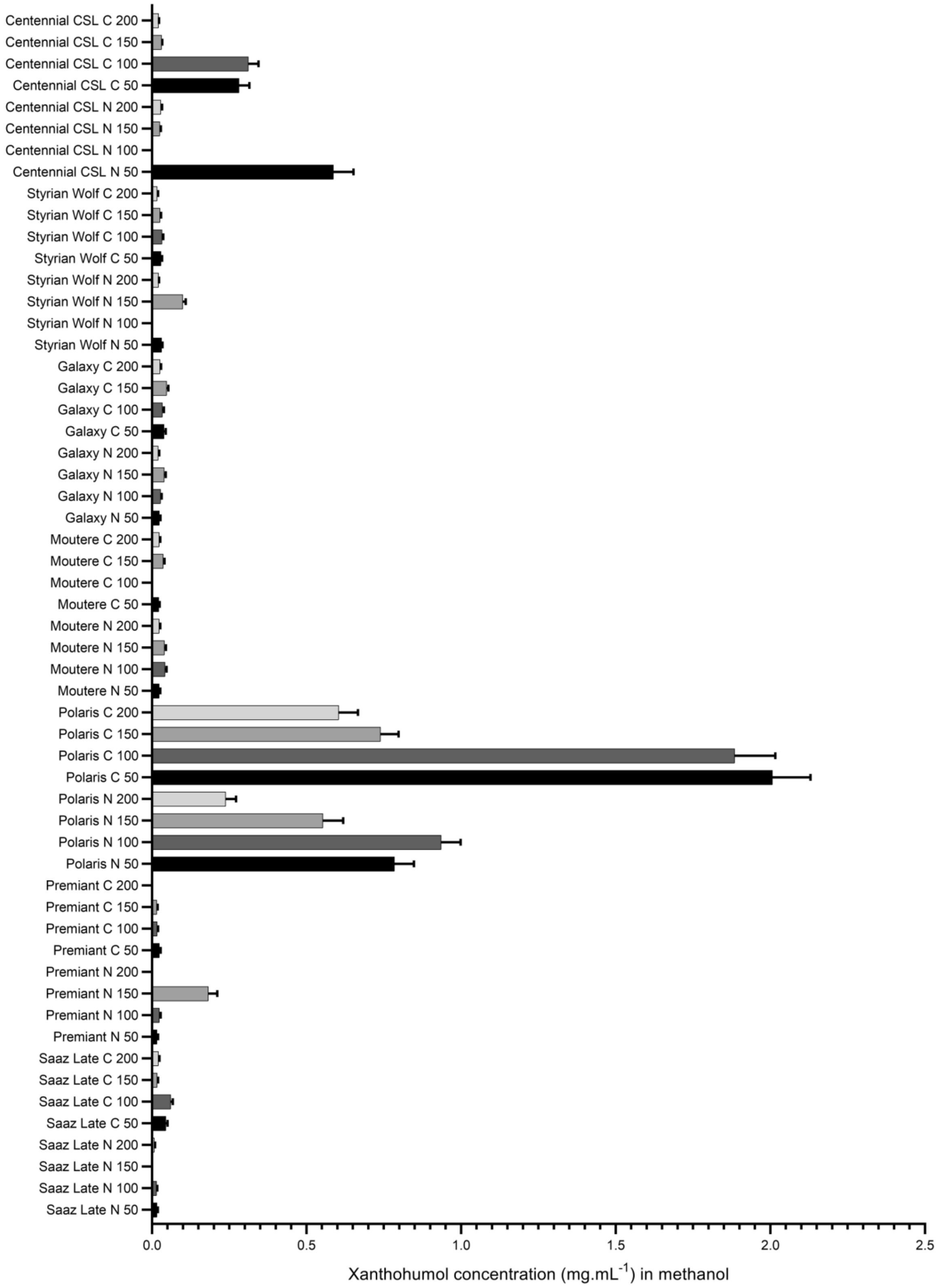

Figure S2. *Xanthohumol extraction profile in methanol.*

Extraction profile of xanthohumol (XN) in hop extracts obtained by ASE using **methanol** as the extraction medium under identical conditions as in Figure S1. Data are expressed as mean ± SD (n = 3). Color intensity corresponds to extraction temperature: the darkest color represents 50 °C, while the lightest color represents 200 °C.

Table S2. Full dataset of isoxanthohumol (IXN) concentrations [mg·mL^{-1}] in hop extracts obtained by ASE under various extraction conditions for all hop varieties.

Variety	Homogenization	Solvent	50 °C	100 °C	150 °C	200 °C
Saaz Late	Mechanical	Ethanol	0.000 ± 0.000	0.000 ± 0.000	0.000 ± 0.000	0.339 ± 0.027
Saaz Late	Cryogenic	Ethanol	0.604 ± 0.051	0.133 ± 0.012	0.034 ± 0.003	0.231 ± 0.027
Premiant	Mechanical	Ethanol	0.001 ± 0.000	0.015 ± 0.002	0.004 ± 0.001	0.097 ± 0.009
Premiant	Cryogenic	Ethanol	0.004 ± 0.001	0.004 ± 0.001	0.005 ± 0.001	0.009 ± 0.002
Centennial (CSL)	Mechanical	Ethanol	0.343 ± 0.027	0.372 ± 0.027	1.024 ± 0.054	1.562 ± 0.105
Centennial (CSL)	Cryogenic	Ethanol	0.000 ± 0.000	0.000 ± 0.000	0.180 ± 0.020	0.290 ± 0.027
Galaxy	Mechanical	Ethanol	0.207 ± 0.023	0.212 ± 0.023	0.044 ± 0.004	0.294 ± 0.027
Galaxy	Cryogenic	Ethanol	0.029 ± 0.003	0.506 ± 0.027	0.540 ± 0.026	1.029 ± 0.055
Styrian Wolf	Mechanical	Ethanol	0.451 ± 0.028	0.321 ± 0.027	0.379 ± 0.028	0.707 ± 0.052
Styrian Wolf	Cryogenic	Ethanol	0.408 ± 0.029	0.245 ± 0.026	0.235 ± 0.026	0.594 ± 0.053
Moutere	Mechanical	Ethanol	0.313 ± 0.027	0.108 ± 0.015	0.031 ± 0.004	0.503 ± 0.035
Moutere	Cryogenic	Ethanol	0.274 ± 0.026	0.053 ± 0.010	0.037 ± 0.004	0.394 ± 0.030
Polaris	Mechanical	Ethanol	0.498 ± 0.029	0.441 ± 0.027	0.419 ± 0.027	0.793 ± 0.054
Polaris	Cryogenic	Ethanol	1.096 ± 0.059	1.731 ± 0.105	1.200 ± 0.077	1.852 ± 0.104
Saaz Late	Mechanical	Methanol	0.009 ± 0.002	0.013 ± 0.003	0.005 ± 0.001	0.007 ± 0.001
Saaz Late	Cryogenic	Methanol	0.296 ± 0.026	0.214 ± 0.024	0.286 ± 0.026	0.098 ± 0.012
Premiant	Mechanical	Methanol	0.000 ± 0.000	0.003 ± 0.001	0.000 ± 0.000	0.003 ± 0.001
Premiant	Cryogenic	Methanol	0.199 ± 0.024	0.168 ± 0.021	0.124 ± 0.015	0.091 ± 0.012
Centennial (CSL)	Mechanical	Methanol	0.048 ± 0.008	0.041 ± 0.008	0.323 ± 0.027	0.452 ± 0.028
Centennial (CSL)	Cryogenic	Methanol	0.029 ± 0.004	0.020 ± 0.003	0.288 ± 0.026	0.345 ± 0.026

Galaxy	Mechanical	Methanol	0.218 ± 0.024	0.197 ± 0.024	0.259 ± 0.027	0.146 ± 0.020
Galaxy	Cryogenic	Methanol	0.167 ± 0.021	0.184 ± 0.022	0.207 ± 0.023	0.079 ± 0.010
Styrian Wolf	Mechanical	Methanol	0.290 ± 0.027	0.333 ± 0.028	0.289 ± 0.027	0.007 ± 0.001
Styrian Wolf	Cryogenic	Methanol	0.194 ± 0.023	0.170 ± 0.020	0.224 ± 0.026	0.006 ± 0.001
Moutere	Mechanical	Methanol	0.001 ± 0.000	0.269 ± 0.027	0.004 ± 0.001	0.403 ± 0.028
Moutere	Cryogenic	Methanol	0.427 ± 0.029	0.140 ± 0.020	0.024 ± 0.003	0.269 ± 0.027
Polaris	Mechanical	Methanol	0.153 ± 0.020	0.102 ± 0.009	0.101 ± 0.009	0.079 ± 0.008
Polaris	Cryogenic	Methanol	0.834 ± 0.053	1.016 ± 0.052	0.791 ± 0.053	0.675 ± 0.051

Values are expressed as mean ± SD (n = 3). ANOVA summary (IXN): $\eta^2 = 0.291$ (Hops), 0.140 (Extractant), 0.050 (Temperature), 0.034 (Homogenization); $p < 0.001$.

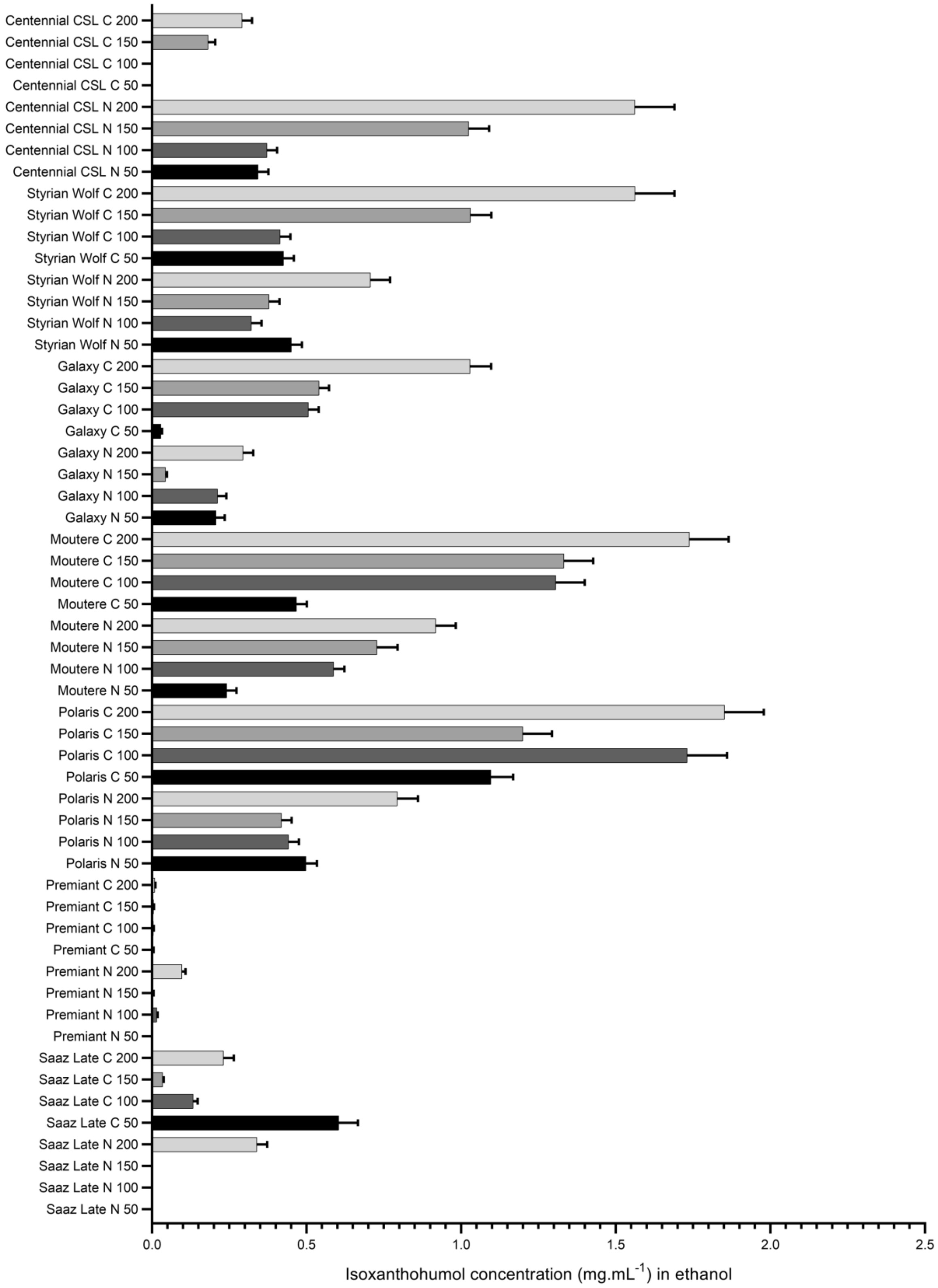

Figure S3. *Isoxanthohumol extraction profile in ethanol.*

Extraction profile of isoxanthohumol (IXN) in hop extracts obtained by ASE using ethanol as the extraction medium at 50–200 °C across all hop varieties. Data are expressed as mean ± SD (n = 3). Color intensity corresponds to extraction temperature: the darkest color represents 50 °C, while the lightest color represents 200 °C.

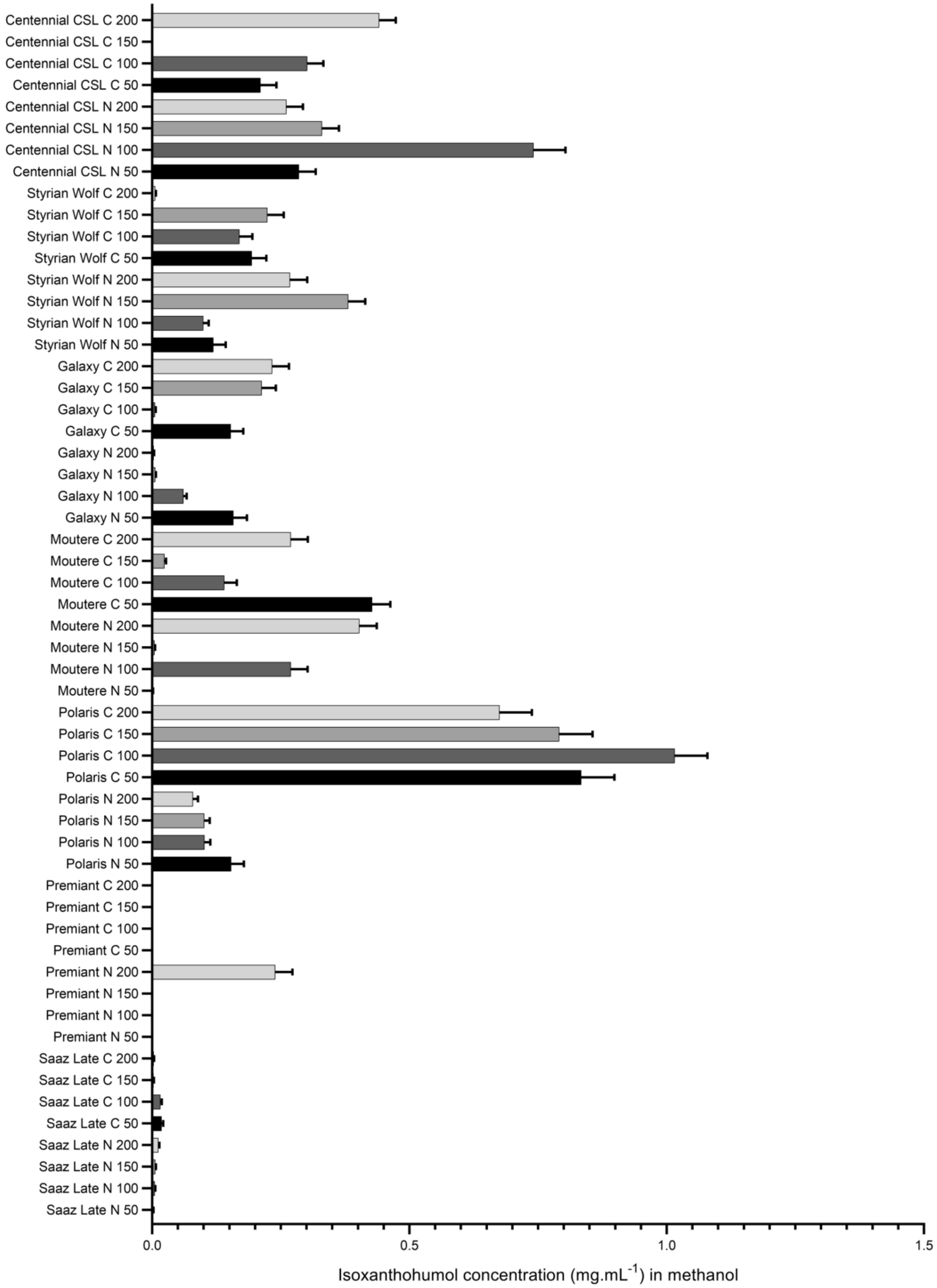

Figure S4. *Isoxanthohumol extraction profile in methanol.*
Extraction profile of isoxanthohumol (IXN) in hop extracts obtained by ASE using methanol under the same experimental conditions as in Figure S3. Data are expressed as mean ± SD (n = 3). Color intensity corresponds to extraction temperature: the darkest color represents 50 °C, while the lightest color represents 200 °C.

Table S3. Full dataset of 8-prenylnaringenin (8-PN) concentrations [mg·mL^{-1}] in hop extracts obtained by ASE under different homogenization, solvent, and temperature conditions for all hop varieties.

Variety	Homogenization	Solvent	50 °C	100 °C	150 °C	200 °C
Saaz Late	Mechanical	Ethanol	0.187 ± 0.020	0.000 ± 0.000	0.000 ± 0.000	0.077 ± 0.007
Saaz Late	Cryogenic	Ethanol	0.122 ± 0.012	0.000 ± 0.000	0.082 ± 0.008	0.095 ± 0.007
Premiant	Mechanical	Ethanol	0.000 ± 0.000	0.000 ± 0.000	0.215 ± 0.020	0.141 ± 0.012
Premiant	Cryogenic	Ethanol	0.312 ± 0.024	0.118 ± 0.011	0.156 ± 0.014	0.161 ± 0.012
Centennial (CSL)	Mechanical	Ethanol	0.087 ± 0.010	0.397 ± 0.028	0.108 ± 0.009	0.102 ± 0.009
Centennial (CSL)	Cryogenic	Ethanol	0.000 ± 0.000	0.000 ± 0.000	0.000 ± 0.000	0.000 ± 0.000
Galaxy	Mechanical	Ethanol	0.000 ± 0.000	0.077 ± 0.007	0.101 ± 0.009	0.000 ± 0.000
Galaxy	Cryogenic	Ethanol	0.080 ± 0.008	0.492 ± 0.028	0.196 ± 0.020	0.260 ± 0.021
Styrian Wolf	Mechanical	Ethanol	0.000 ± 0.000	0.423 ± 0.028	0.122 ± 0.011	0.265 ± 0.021
Styrian Wolf	Cryogenic	Ethanol	0.000 ± 0.000	0.455 ± 0.029	0.152 ± 0.013	0.235 ± 0.020
Moutere	Mechanical	Ethanol	0.000 ± 0.000	0.201 ± 0.020	0.140 ± 0.013	0.195 ± 0.016
Moutere	Cryogenic	Ethanol	0.000 ± 0.000	0.538 ± 0.030	0.230 ± 0.020	0.279 ± 0.021
Polaris	Mechanical	Ethanol	0.000 ± 0.000	0.134 ± 0.012	0.262 ± 0.021	0.450 ± 0.028
Polaris	Cryogenic	Ethanol	0.101 ± 0.009	0.590 ± 0.029	0.506 ± 0.028	0.363 ± 0.024
Saaz Late	Mechanical	Methanol	0.000 ± 0.000	0.000 ± 0.000	0.000 ± 0.000	0.000 ± 0.000
Saaz Late	Cryogenic	Methanol	0.000 ± 0.000	0.000 ± 0.000	0.000 ± 0.000	0.000 ± 0.000
Premiant	Mechanical	Methanol	0.000 ± 0.000	0.000 ± 0.000	0.000 ± 0.000	0.000 ± 0.000
Premiant	Cryogenic	Methanol	0.000 ± 0.000	0.000 ± 0.000	0.000 ± 0.000	0.000 ± 0.000
Centennial (CSL)	Mechanical	Methanol	0.000 ± 0.000	0.000 ± 0.000	0.000 ± 0.000	0.000 ± 0.000
Centennial (CSL)	Cryogenic	Methanol	0.000 ± 0.000	0.000 ± 0.000	0.000 ± 0.000	0.000 ± 0.000

Galaxy	Mechanical	Methanol	0.000 ± 0.000	0.000 ± 0.000	0.000 ± 0.000	0.000 ± 0.000
Galaxy	Cryogenic	Methanol	0.000 ± 0.000	0.000 ± 0.000	0.000 ± 0.000	0.000 ± 0.000
Styrian Wolf	Mechanical	Methanol	0.000 ± 0.000	0.000 ± 0.000	0.000 ± 0.000	0.000 ± 0.000
Styrian Wolf	Cryogenic	Methanol	0.124 ± 0.010	0.000 ± 0.000	0.170 ± 0.013	0.143 ± 0.011
Moutere	Mechanical	Methanol	0.000 ± 0.000	0.000 ± 0.000	0.000 ± 0.000	0.000 ± 0.000
Moutere	Cryogenic	Methanol	0.092 ± 0.008	0.087 ± 0.008	0.095 ± 0.008	0.000 ± 0.000
Polaris	Mechanical	Methanol	0.000 ± 0.000	0.233 ± 0.022	0.000 ± 0.000	0.086 ± 0.008
Polaris	Cryogenic	Methanol	0.000 ± 0.000	0.000 ± 0.000	0.240 ± 0.021	0.105 ± 0.009

Values are expressed as mean ± SD ($n = 3$). ANOVA summary (8-PN): $\eta^2 = 0.174$ (Extractant), 0.128 (Hops), 0.046 (Temperature), 0.082 (Homogenization); $p < 0.001$.

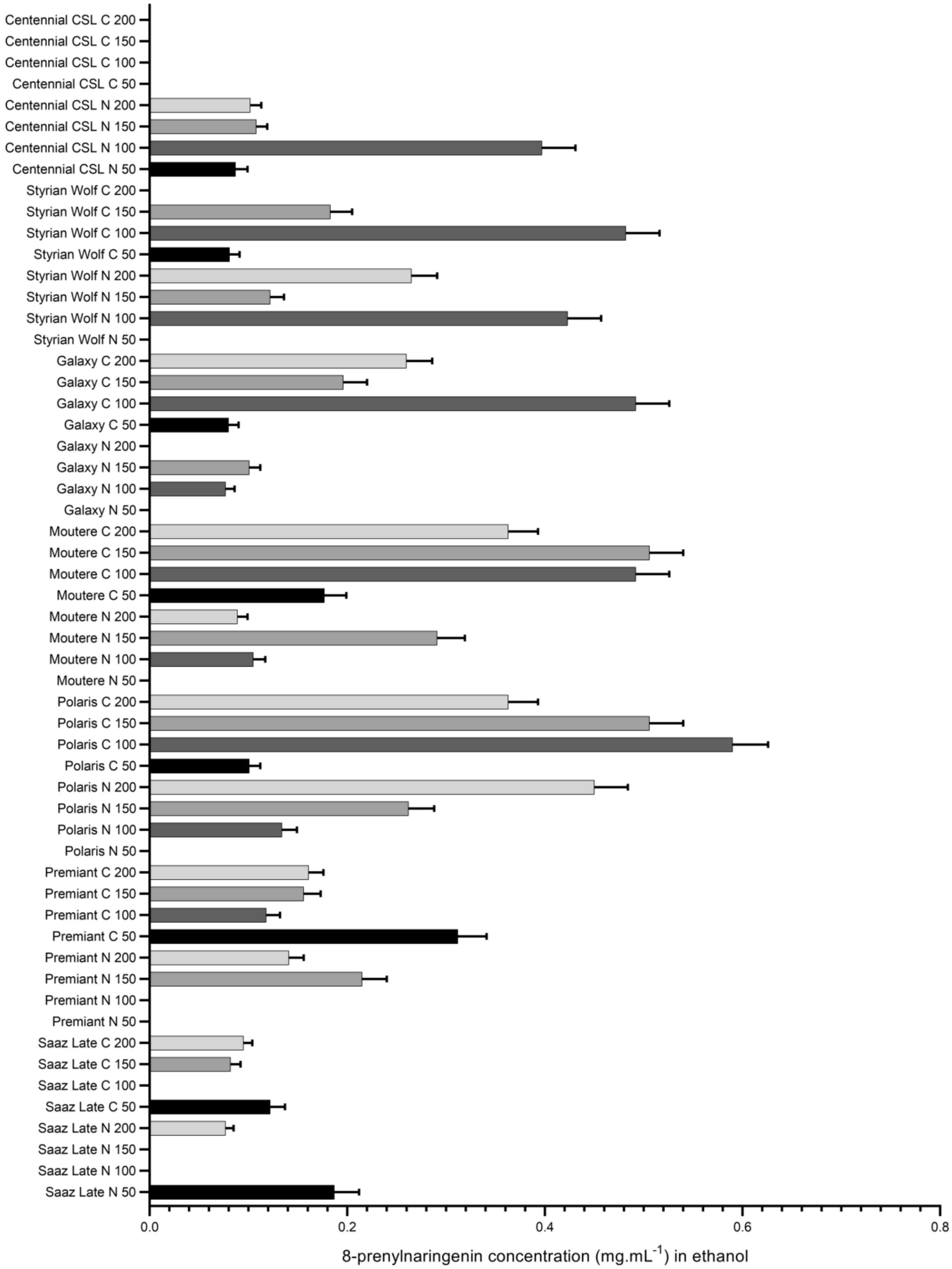

Figure S5. *8-Prenylnaringenin extraction profile in ethanol.*
Extraction profile of 8-prenylnaringenin (8-PN) in hop extracts obtained by ASE using ethanol as the extraction medium at 50–200 °C. Data are expressed as mean ± SD (n = 3). Color intensity corresponds to extraction temperature: the darkest color represents 50 °C, while the lightest color represents 200 °C.

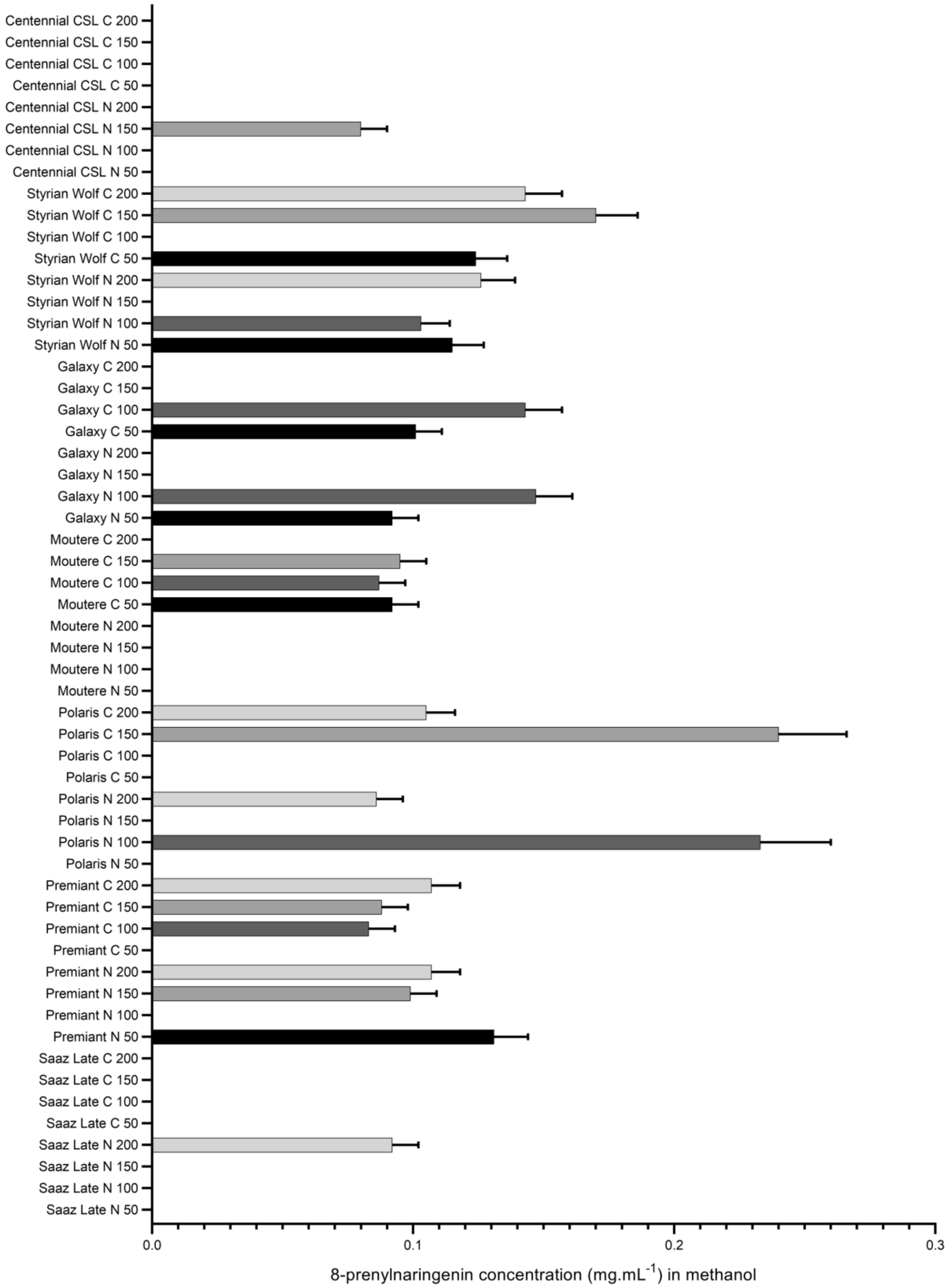

Figure S6. *8-Prenylnaringenin extraction profile in methanol.*
Extraction profile of 8-prenylnaringenin (8-PN) in hop extracts obtained by ASE using methanol under identical conditions as in Figure S5. Data are expressed as mean ± SD (n = 3). Color intensity corresponds to extraction temperature: the darkest color represents 50 °C, while the lightest color represents 200 °C.

Multifactorial ANOVA was performed to evaluate the significance of individual factors and their interactions on the extraction yield of xanthohumol (XN), isoxanthohumol (IXN), and 8-prenylnaringenin (8-PN).
The results summarize the main effects of hop variety, solvent type, extraction temperature, and homogenization method, as well as their interactions.
All factors were significant at $p < 0.001$, confirming the combined influence of chemical and technological parameters on the extraction efficiency of prenylated flavonoids.
Detailed statistical outcomes are presented in Tables S4–S6.

Table S4. Results of multifactor ANOVA for xanthohumol (XN).

Source of Variation	df	F-value	p-value	η^2	Partial η^2
Hops variety	6	929.3	<0.001	0.323	0.321
Solvent	1	784.6	<0.001	0.266	0.264
Temperature	3	102.8	<0.001	0.018	0.017
Homogenization	1	89.1	<0.001	0.020	0.019
Hops × Solvent	6	512.5	<0.001	0.185	0.183
Hops × Homogenization	6	71.2	<0.001	0.040	0.038
Error	224	—	—	—	—

ANOVA summary for XN showing the influence of variety, solvent, temperature, and homogenization on extraction yield. All factors were statistically significant ($p < 0.001$).

Table S5. Results of multifactor ANOVA for isoxanthohumol (IXN).

Source of Variation	df	F-value	p-value	η^2	Partial η^2
Hops variety	6	299.5	<0.001	0.291	0.288
Solvent	1	243.4	<0.001	0.140	0.138
Temperature	3	58.7	<0.001	0.050	0.048
Homogenization	1	42.8	<0.001	0.034	0.033
Hops × Solvent	6	127.3	<0.001	0.076	0.075
Hops × Homogenization	6	98.6	<0.001	0.164	0.162
Error	224	—	—	—	—

ANOVA summary for IXN showing the significant effects of extraction parameters and their interactions on the isomerization efficiency from XN.

Table S6. Results of multifactor ANOVA for 8-prenylnaringenin (8-PN).

Source of Variation	df	F-value	p-value	η^2	Partial η^2
Hops variety	6	256.7	<0.001	0.128	0.126

Solvent	1	289.2	<0.001	0.174	0.172
Temperature	3	62.4	<0.001	0.046	0.044
Homogenization	1	54.1	<0.001	0.082	0.081
Hops × Temperature	6	114.3	<0.001	0.128	0.126
Hops × Homogenization	6	93.9	<0.001	0.082	0.081
Error	224	—	—	—	—

ANOVA summary for 8-PN confirming significant effects of solvent type, hop variety, temperature, and homogenization on extraction yield ($p < 0.001$).
